# A geostatistical analysis of the association between armed conflicts and *Plasmodium falciparum* malaria in Africa, 1997–2010

**DOI:** 10.1186/s12936-015-1024-5

**Published:** 2015-12-16

**Authors:** Luigi Sedda, Qiuyin Qi, Andrew J. Tatem

**Affiliations:** CHICAS, Lancaster Medical School, Lancaster University, Furness Building, Lancaster, LA1 4YG UK; Department of Geography, University of Florida, Gainesville, FL 32611-7315 USA; Fogarty International Center, National Institutes of Health, Bethesda, MD 20892 USA; Flowminder Foundation, Roslagsgatan 17, 113 55 Stockholm, Sweden; Geography and Environment, University of Southampton, University Road, Southampton, SO17 1BJ UK

**Keywords:** *Plasmodium falciparum* parasite rate 2–10, Conflict density, Violence, Variogram, Malaria control

## Abstract

**Background:**

The absence of conflict in a country has been cited as a crucial factor affecting the operational feasibility of achieving malaria control and elimination, yet mixed evidence exists on the influence that conflicts have had on malaria transmission. Over the past two decades, Africa has seen substantial numbers of armed conflicts of varying length and scale, creating conditions that can disrupt control efforts and impact malaria transmission. However, very few studies have quantitatively assessed the associations between conflicts and malaria transmission, particularly in a consistent way across multiple countries.

**Methods:**

In this analysis an explicit geostatistical, autoregressive, mixed model is employed to quantitatively assess the association between conflicts and variations in *Plasmodium falciparum* parasite prevalence across a 13-year period in sub-Saharan Africa.

**Results:**

Analyses of geolocated, malaria prevalence survey variations against armed conflict data in general showed a wide, but short-lived impact of conflict events geographically. The number of countries with decreased *P. falciparum* parasite prevalence (17) is larger than the number of countries with increased transmission (12), and notably, some of the countries with the highest transmission pre-conflict were still found with lower transmission post-conflict. For four countries, there were no significant changes in parasite prevalence. Finally, distance from conflicts, duration of conflicts, violence of conflict, and number of conflicts were significant components in the model explaining the changes in *P. falciparum* parasite rate.

**Conclusions:**

The results suggest that the maintenance of intervention coverage and provision of healthcare in conflict situations to protect vulnerable populations can maintain gains in even the most difficult of circumstances, and that conflict does not represent a substantial barrier to elimination goals.

## Background

The number of ongoing armed conflicts in the world has declined steadily through the 1990s and early 2000s, with a 40 % reduction from peak years shortly after the end of the Cold War [1]. However, this trend ended in the mid-2000s and the annual frequency of major armed conflicts has stabilized at around 35 in recent years, with most concentrated in Asia and Africa [2]. Over the past two decades, at least 20 African countries have been involved in armed conflicts of various types and levels of intensity (e.g., civil wars, interstate wars and violence against civilians) [2]. These conflicts continue to exert assorted detrimental effects, including many deaths, substantial economic losses and large numbers of forcibly displaced people [3, 4]. It is estimated that armed conflicts cost Africa approximately $18 bn per year and have shrunken each conflict-afflicted African nation’s economy by 15 % on average since 1990 [5]. By the end of 2011, Africa hosted more than a quarter (2.7 million) of the world’s 10.4 million refugees [6] and one-third (9.7 million) of the world’s 26.4 million internally displaced persons [7], while only constituting 15 % of the world total population [8].

The challenges that armed conflicts pose on public health are widely acknowledged, not only the direct injuries and deaths among military personnel and civilians, but also the indirect effects on the physical and socio-economic environments that exacerbate morbidity and mortality [9–11]. Infectious disease, including malaria, is often a significant health problem during and after conflicts [12], as multiple risk factors flourish that enhance disease emergence and transmission, including displacement of large non-immune populations to endemic areas [13, 14], resettlement of refugees to deteriorated environments that favour vector breeding (e.g., inadequate sanitation, marginal land) [9], disruption of disease control programmes, breakdown of health systems [15, 16], and impeded access to populations for timely delivery of medical supplies [17–19]. For example, the civil war in Tajikistan during 1992–1993 led to an increase in annually reported malaria cases from 200 in 1992 to almost 30,000 in 1997 [20].

Several studies have examined the effects of armed conflicts on malaria transmission [21–24] and explored barriers and strategies for malaria interventions and control in conflict situations [18, 25]. The majority of theories and findings suggest that armed conflicts are associated with increased malaria risk [17, 18, 21, 26, 27]. However, there are also studies indicating a negative association between level of conflicts and malaria risk [22]; describing successful malaria control in conflict-affected regions, such as Sri Lanka, which has almost eliminated malaria despite nearly 30 years of civil war [25, 28]. Generally, most studies examining the relationship between armed conflicts and malaria transmission are descriptive or limited to individual countries. As such, there is a lack of research investigating the association between conflicts and malaria quantitatively over large areas.

**Fig. 1:**
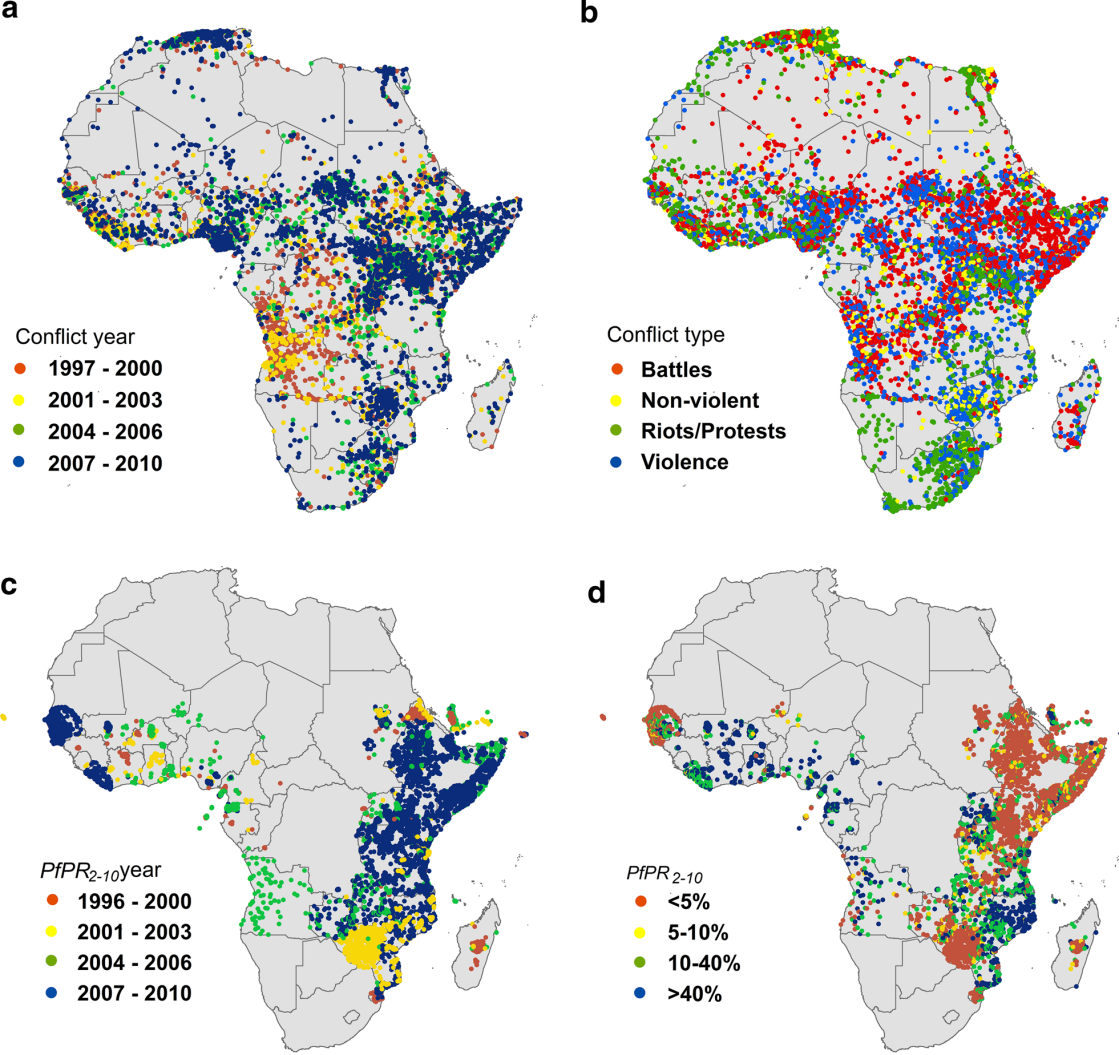
Datasets used in the analyses. Recorded conflict events 1997–2010 in Africa for different years (**a**) and type (**b**); *P. falciparum* parasite rate (*Pf*PR) surveys 1997–2010, standardized to the 2–10 years age group for different years (**c**) and type (**d**)

Given declines in malaria prevalence in Africa [29], a renewed international focus on malaria eradication, and that the absence of internal and external conflicts can be a crucial factor affecting the operational feasibility of malaria elimination [30], there is a need to understand and quantify the relationships between armed conflicts and malaria by analysing the influence of the proximity of conflicts (in space and time) on the temporal variability of malaria transmission. To explore if significant increases in malaria transmission during or after conflicts have been seen in Africa, the most comprehensive geo-referenced databases of *Plasmodium falciparum* parasite rate (*Pf*PR) surveys [31] (Fig. 1c, d) and armed conflict events (Fig. 1a, b) were integrated for the 1997–2010 period and their spatial relationships investigated.

## Methods

### Data

Geo-located *Pf*PR community surveys across sub-Saharan Africa were obtained from the Malaria Atlas Project database [32, 33] (Fig. 1d). Among the various metrics of malaria transmission, PR is the most commonly reported and reliable metric for *P. falciparum* malaria endemicity [34] and sensitive across a broad range of the transmission spectrum [35]. The logistically intensive process of searching for, identifying and geo-referencing the *Pf*PR surveys has been documented elsewhere [33, 36], with all of the surveys geo-located and not duplicated within 3 months at the same site. As *Pf*PR follows a pattern related to age and is generally reported across different age ranges [37, 38], an algorithm described by Smith et al. [39] was applied to standardize the values of *Pf*PR to a single and epidemiologically important age group (2–10 years). Of the surveys in Africa, in particular, the majority were conducted after 2000 (79 %, Fig. 1c) and diagnosed through microscopy (71 %). The sample size of these surveys varies and more than half of them (52 %) are larger than 50. At the time of analysis, a total of 15,213 *Pf*PR surveys undertaken between 1997 and 2010 were available for sub-Saharan Africa [33].

The conflict data were obtained from the Armed Conflict Location and Event Dataset (ACLED) [40], which assembles and codes reported violent events in unstable and warring states and has been shown to be robust for continental and regional studies [41, 42]. This dataset, which covers all of the countries in Africa (Fig. 1a), provides detailed information on the dates, locations, event type, groups involved, information sources, and fatalities for armed conflicts [40]. Specifically, it focuses on tracking rebel, militia and government activities, identifying territorial transfers and collecting information on rioting, protests and non-violent events [40]. By 31 December, 2010, 48,261 events in Africa had been recorded by ACLED with 44 % being battles between government, rebels and militias, 36 % violence against civilians, 14 % riots and protests, and 6 % non-violent events (Fig. 1b). Each entry in this disaggregated dataset is ‘atomic’, in the sense that events which took place over multiple days are recorded as consecutive events on a specific day and in an exact location [40].

## Methods

### General model

An autoregressive, additive, geostatistical, linear mixed model was applied to the malaria surveys and the surrounding conflict locations. Autoregressive components are often used in spatiotemporal analyses of malaria prevalence [43, 44], and enable relative (to its initial value) measures of the *Pf*PR variation to be produced. Spatial and spatiotemporal autocorrelation has been found to be significant in other conflicts [11, 42, 45] and malaria studies [31, 44, 46].

A preliminary analysis was carried out to test if the differences in *Pf*PR (in the 2–10 age range) before (*Pf*PR_b_) and after (*Pf*PR_a_) conflicts, *ΔP*, were more accurately explained by the model than using *Pf*PR_a_ as the dependent variable. The results showed that using *ΔP* increased the explanatory power of the model by 12 %. In addition, fitting *ΔP* enabled removal of some uncertainties due to the transformation of *Pf*PR_a_ to *ΔP* in the post-modelling stage. Therefore, for each conflict location, *ΔP* was calculated as:

1a $$ \varDelta P = Pf{\text{PR}}_{\text{b}} - Pf{\text{PR}}_{\text{a}} - S_{m} $$

1b $$ S_{m} = A + \left( {B\sin \left( {\theta \cdot m} \right)} \right)\quad \quad m = 1, \ldots ,T $$

where *Pf*PR_a_ and *Pf*PR_b_ are obtained from each malaria survey within 5° and 10 months from the conflict location. In other words only *Pf*PR collected in different times at the same location are considered (6205 malaria surveys). In fact, taking into account single surveys (*Pf*PR surveyed only once in a location) and averaging their values with those from other surveys within 5° and 10 months from a conflict event, reduced the explanatory power of the model (−26 %).

The values of 5° (parameter ${\varphi}$) and 10 months (parameter *ρ*) were obtained from a Monte Carlo simulation in which, at each iteration, 20 % of the *ΔP* values were randomized in space and time. At each of the 10,000 iterations, the gamma variance for each combination of spatial and temporal lags (spatial lags, ${\varphi}$, spanning from 0.1° to 10° between conflict location and malaria surveys; and temporal lags, *ρ*, spanning from 1 to 100 months between conflict starting date, *c*, and the time of the malaria surveys) were calculated by fitting the γ-variances (known as experimental variogram):

2 $$ {{\gamma \left( {\varphi ,\rho } \right) = \frac{1}{2}\frac{1}{n\left( \varphi \right)}\frac{1}{{n\left( {c + \rho } \right)}}\frac{1}{{n\left( {c - \rho } \right)}}\sum\limits_{i = 1}^{n\left( \varphi \right)} {\sum\limits_{ii = 1}^{{n\left( {c + \rho } \right)}} {\sum\limits_{iii = 1}^{{n\left( {c - \rho } \right)}} {\left( {PfPR_{i,ii} - PfPR_{i,iii} } \right)}^{2} } } }} $$

with a non-separable, spatiotemporal, exponential function and applying the non-linear minimization method *nml* [47]:

3 $$ {{\hat{\gamma }\left( {\varphi ,\rho } \right) = \exp \left( { - \frac{d}{\varphi } + \frac{d}{\varphi }\frac{h}{\rho } - \frac{h}{\rho }} \right)}} $$

where *d* and *h* are the spatial Haversine distance and the temporal distance, respectively, between conflicts and malaria settings. Five degrees and 10 months are the average spatial and temporal lags obtained from the Monte Carlo simulation. Equation [2] differs from the canonical equation of the variogram in the lag parameters, as they express the spatial and temporal distances between malaria survey locations and the conflict and not the distance between malaria surveys.

The parameter *S*_*m*_ in Eq. (1b) is the seasonality in the *Pf*PR modelled as sinusoidal function of the scaling parameter *A*, the parameter controlling the amplitude (*B*) and the parameter controlling the phase (*θ*); finally, *m* is the moment along the time series of total length *T*. To obtain *S*_*m*_, the average monthly *Pf*PR from 1997 to 2010 was fitted with a sinusoidal curve (1b). The parameters *A* = 0.31, *B* = 0.1 and *θ* = 1.8 were obtained through applying a least square method to the time series. While the average correction is only 0.04 of the prevalence rate, the use of the de-seasonalized data in Eq. (4a) improved the Akaike Information Criterion (AIC) from −17,356 to −19,516.

A method for accounting for the effects of seasonality was implemented in order to avoid the situation where the period before and after conflict may include a seasonal effect. For example, if *Pf*PR tends to be higher due to seasonality when conflict occurs and lower when measured ‘after conflict’ then the outcome (a measurable decline in *Pf*PR) may simply reflect seasonal effects. Other methods, i.e., the use of covariates in order to simulate seasonality (i.e., precipitation and temperature) may be useful [48], however, this does not solve intrinsic periodicities in endemic-malaria countries, and requires user-defined qualitative (i.e., spatial scale of the seasonality for pre-defined regions) and quantitative parameterization of the model. The complexity of modelling malaria seasonality in Africa, in terms of the time of the year, amplitude and phase is discussed elsewhere [49]. Global, rather than local, corrections for the periodicities in the data are typically applied [43, 44], but more often the seasonal component is not modelled or removed in national or sub-national prevalence mapping studies [50]. Finally, the use of an explicit seasonality component, rather than including the seasonality in the covariance function is due to the use of a correlation matrix dependent on the distance from conflict settlings to malaria surveys and not from malaria surveys to malaria surveys (see below term $\hat{\gamma }$ in Eq. 4c).

The *Pf*PR differences at each conflict location and malaria survey were fitted using an additive, geostatistical, linear mixed model containing an autoregressive component (*PfPR*_*b*_); a matrix of covariates **X** (fixed effects); a matrix of random effects **W**; a spatiotemporal correlation effect, **Z**; and an error component *ε*:

4a $$ \Delta P_{q,t} = \beta_{0} PfPR_{b(q,t)} + \beta_{1} X + bW + Z + \varepsilon $$

4b $$ b\sim N\left( {0,\varSigma_{b} } \right) $$

4c $$ Z \sim N\left( {0,\sigma_{z}^{2} \hat{\gamma }\left( {\varphi ,\rho } \right)} \right) $$

4d $$ \varepsilon \sim N\left( {0,\sigma_{e}^{2} {\rm I}} \right) $$

4e $$ \Delta P_{q,t} \sim N\left( {\beta_{0} PfPR_{b(q,t)} + \beta_{1} X,W\varSigma_{b} W^{T} + \sigma_{z}^{2} \hat{\gamma }\left( {\varphi ,\rho } \right) + \sigma_{e}^{2} {\rm I}} \right) $$

4f $$ \varSigma_{b} = \sigma_{b}^{2} {\rm I} $$

where the subscripts *q* and *t* indicate the location and the time of the conflict event, respectively; *β*_*0*_ and *β*_*1*_ are the regression coefficients for *Pf*PR_b_ and (a vector of coefficients) for **X**, respectively; **b** is a one column vector of aspatial normally distributed random effects with mean zero and covariance matrix **Σ**_b_, given by the product of the variance $\sigma_{b}^{2}$ and the identity matrix **I** [51]; **Z** is a one column vector with spatiotemporal normally distributed random effects with mean zero and a covariance matrix given by the product of the spatial variance, $\sigma_{z}^{2}$, and the correlation matrix, $\hat{\gamma }$. As shown above, $\hat{\gamma }$ is expressed as a function of the spatial correlation parameter, ${\varphi}$, defining the spatial range of *ΔP* from the conflict location; and the temporal correlation, *ρ*, defining the temporal range of Δ*P* from the conflict location (Eq. 3). Finally, $\varepsilon$ is the independent and identically normally distributed error, with error variance $\sigma_{e}^{2}$**I**. The covariates in **X** are: number of conflicts experienced at the malaria survey location, its distance from conflicts and duration of the conflicts at the conflict location. In addition, the typology of conflicts is considered in **X** in form of dummy variables (1, Battle-Government regains territory; 2, Battle-no change of territory; 3, Battle-non-state actor overtakes territory; 4, Headquarters or base established; 5, Non-violent activity by a conflict actor; 6, Non-violent transfer of territory; 7, Riots/protests; 8, Violence against civilians; see [52] for conflict-type definition). Environmental and socio-economic variables are not taken into account because this analysis focuses only on the relationships between spatial and temporal dimensions in conflicts and malaria prevalence surveys (point to point analyses). Including non-conflict variables can likely explain additional variance in the differences in *Pf*PR but it is unlikely to alter the contribution of the conflict variables in prevalence changes. In order to account for country characteristics, the random effect is the country of the malaria survey location and the macro area. For the latter, a dichotomous variable with values ‘East Africa’ and ‘West Africa’ was employed due to the differences in prevalence sample sizes [53].

Model (4a) was the best model from various alternatives where different types of dependent variables (*Pf*PR, at malaria locations and conflict locations), fixed effects (e.g., longitude and latitude of conflict, longitude and latitude of malaria surveys), spatiotemporal effects (e.g., without **Z**, only spatial, using parameters obtained from the canonical variogram of malaria or from the variogram of conflicts) and random effects (only country or macro-area, no random effects) were considered.

### Validation

To evaluate the accuracy of the model, it was re-run keeping out 20 % of the records for which it was estimated the $\Delta \hat{P}$ and compared with real *ΔP* through the following statistics:

1. Mean error
  5 $$ ME = \frac{1}{Q}\sum\limits_{q = 1}^{Q} {\Delta P_{q} - \Delta \hat{P}_{q} } $$
2. Mean squared error
  6 $$ MSE = \frac{1}{Q}\sum\limits_{q = 1}^{Q} {\left( {\Delta P_{q} - \Delta \hat{P}_{q} } \right)^{2} } $$
3. Mean squared deviation ratio
  7 $$ MSDR = \frac{1}{Q}\sum\limits_{q = 1}^{Q} {\frac{{\left( {\Delta P_{q} - \Delta \hat{P}_{q} } \right)^{2} }}{{\left( {\sigma_{k}^{2} } \right)_{q} }}} $$
  where $\left( {\sigma_{k}^{2} } \right)_{s}$ is the kriging variance at location *q*. The ideal values for ME, MSE and (1 − MSDR) are zero. For the latter, this means that MSE equals the kriging variance (hence MSDR = 1).

### $\Delta \hat{P}$ Africa map

**Fig. 2:**
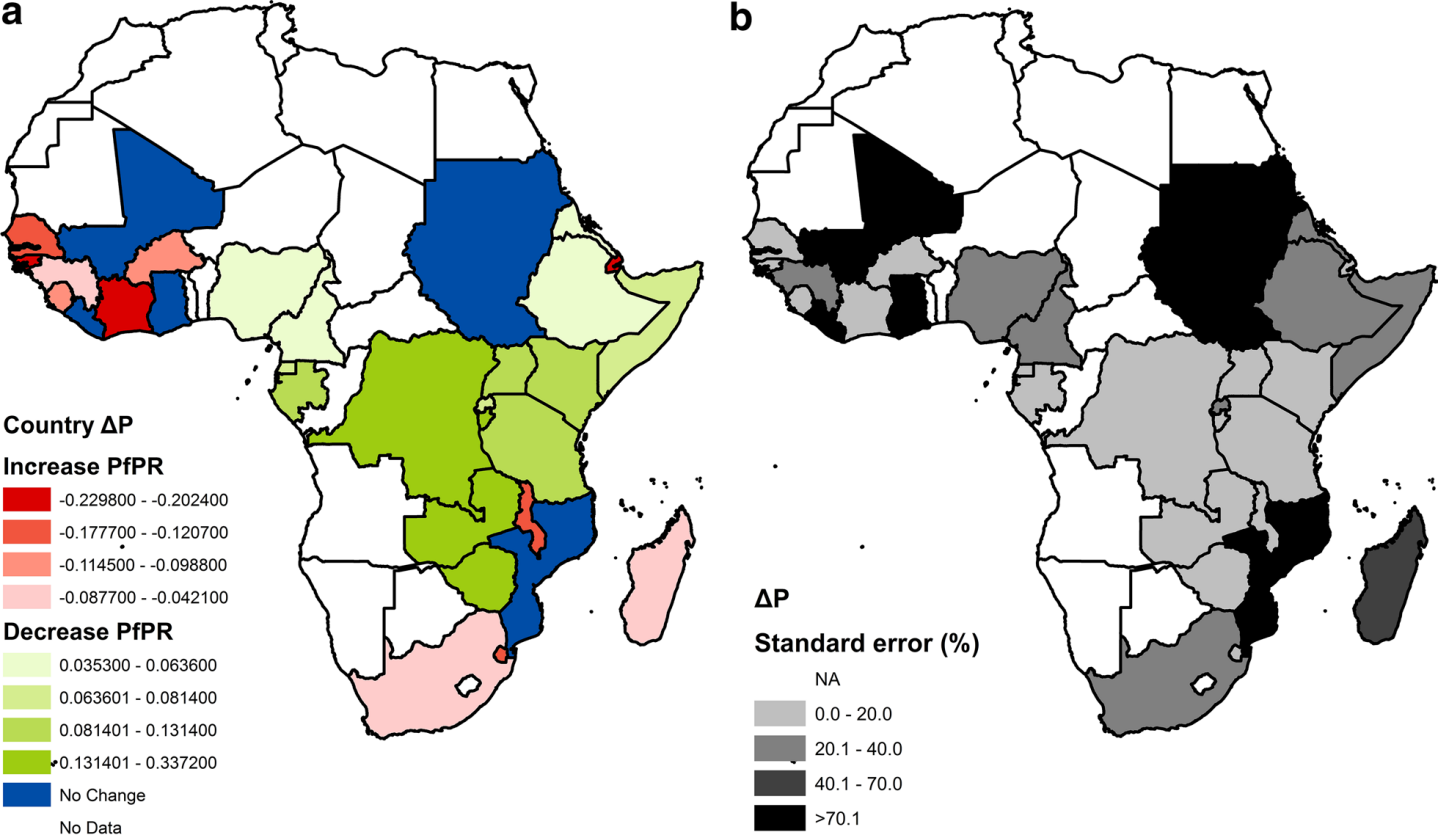
Per-country estimated change in *Pf*PR from before to after conflict events over the 1997–2010 period, $\Delta \hat{P}$. **a** Estimated $\Delta \hat{P}$, larger positive/negative values indicate larger decrease (if positive) or increase (if negative) in *Pf*PR after conflict. **b** Standard error in $\Delta \hat{P}$ estimation; where the standard error is larger than 100 % of the estimated $\Delta \hat{P}$, the country decrease/increase in *Pf*PR is not considered (Liberia, Mali, Mozambique, and Sudan)

A country-level measurement of $\Delta \hat{P}$ was produced by averaging the fitted $\Delta \hat{P}$ values for each country (Fig. 2a). The countries are classified in the following four categories: places where *Pf*PR was predicted to be increasing in relation to conflict events ($\Delta \hat{P}$ + standard error < 0); places where *Pf*PR was predicted to be decreasing in relation to conflict events ($\Delta \hat{P}$ − standard error > 0); and places where $\Delta \hat{P}$ estimations are affected by a large standard error and hence cannot be categorized. A fourth category is for those countries which data are not enough for estimation. Here, the standard error represented the uncertainty in the values of the covariance and error noise components.

## Results

When considering Africa as a whole, a pattern emerged with west Sub-Saharan Africa and southeast Africa showing increases in *Pf*PR following conflicts, and the greater Horn of Africa (with the exception of Djibouti) and Central Africa showing decreases in *Pf*PR after conflict (Fig. 2; Table 1). Even for some of the most conflict-affected countries, such as Zimbabwe, Somalia, Democratic Republic of Congo, and Sudan, significantly lower prevalences were evident in the months after conflict events, compared to before. In 17 out of the 33 sub-Saharan African countries for which *Pf*PR post-conflict estimation was possible and significant, decreases in prevalence were evident when comparing pre- to post-conflict levels (Table 1). For the other four countries, the model was not able to find significant changes in P*f*PR after conflict. Assessments of existing evidence were not all positive. Thirty-six per cent of the countries (generally those affected by high-medium transmission levels) did not show an immediate ability to bounce back from periods of major conflict and showed an increase in *Pf*PR post-conflict (Table 1). Interestingly, large increases/decreases were always associated with low uncertainty (Fig. 2b), however, even when the uncertainty was large the estimates were still significantly different from zero, with the exception of Ghana, Liberia, Mali, and Mozambique.

**Table 1:** Averaged-country differences in *Pf*PR before (*Pf*PR_b_) and after (*Pf*PR_a_) conflicts (diff) and its model estimation (est diff) and model standard errors (stand error)

Country	Diff	*Pf*PR_b_	*Pf*PR_a_	Est diff	Stand error
Zimbabwe	0.321	0.532	0.211	0.337	0.015
Zambia	0.280	0.547	0.267	0.272	0.014
Burundi	0.144	0.383	0.239	0.133	0.011
Congo (Democratic Republic of the)	0.136	0.341	0.205	0.140	0.013
Kenya	0.129	0.340	0.212	0.131	0.014
Uganda	0.113	0.355	0.242	0.099	0.014
Tanzania	0.109	0.258	0.150	0.109	0.015
Rwanda	0.085	0.308	0.223	0.067	0.014
Somalia	0.078	0.106	0.028	0.080	0.017
Equatorial Guinea	0.078	0.174	0.097	0.081	0.013
Sao Tome and Principe	0.077	0.131	0.054	0.077	0.014
Nigeria	0.065	0.165	0.101	0.063	0.014
Gabon	0.049	0.073	0.024	0.086	0.012
Ethiopia	0.049	0.064	0.015	0.047	0.015
Eritrea	0.039	0.058	0.019	0.035	0.012
Ghana	0.035	0.706	0.671	−0.011	0.020
Cameroon	0.033	0.229	0.196	0.044	0.010
Sudan	0.006	0.035	0.029	0.006	0.016
Mali	−0.008	0.375	0.382	−0.009	0.014
Mozambique	−0.009	0.278	0.287	−0.001	0.018
Malawi	−0.031	0.358	0.389	−0.121	0.014
Madagascar	−0.039	0.196	0.235	−0.042	0.027
Liberia	−0.046	0.319	0.365	0.011	0.012
South Africa	−0.057	0.283	0.340	−0.087	0.020
Djibouti	−0.089	0.013	0.102	−0.208	0.008
Swaziland	−0.093	0.293	0.386	−0.165	0.017
Burkina Faso	−0.101	0.595	0.696	−0.102	0.013
The Gambia	−0.102	0.136	0.239	−0.098	0.016
Guinea	−0.105	0.210	0.315	−0.066	0.014
Sierra Leone	−0.125	0.210	0.335	−0.114	0.012
Senegal	−0.184	0.083	0.267	−0.177	0.016
Cote D’Ivoire	−0.184	0.379	0.563	−0.202	0.016
Guinea-Bissau	−0.199	0.115	0.313	−0.229	0.011

Thus, what are the determinants of this *ΔP* pattern? The applied model, for which accuracy was high (validation statistics returned a ME = −0.0007, MSE = 0.002 and 1 − MSDR = 0.12), suggests that increases in *ΔP* (i.e., a decrease in *Pf*PR) are associated with lower numbers of conflicts around the malaria surveys, shorter duration of conflicts and longer distance from conflict locations. Regarding the typology of the conflict, the absence of violence against civilians, riots/protests, battles with no change of territory, and non-violent transfer of territory (Table 2) are associated with decrease in post-conflict *Pf*PR, while battles in which government retain territory, non-violent activity by a conflict actor, headquarters and base established, and battles with non-state actors overtaking territory (categories defined by ACLED [52]) were not significantly correlated with changes in *Pf*PR. Therefore, the four types of conflicts that have caused 94 % of deaths from all types of armed conflicts (93 % just violence against civilians and riots/protests), are also those that are influencing the variations in *Pf*PR.

**Table 2:** Estimated coefficients for the independent variables used into the model

Coefficients	Estimate	Standard error	*p* value
*Pf*PR_b_	0.78580	0.00587	<0.001
Number of conflicts	−0.00008	<10^−6^	<0.001
Distance from conflict	0.00142	0.00086	<0.001
Duration of the conflict	−0.00002	0.00001	<0.001
Violence against civilians	−0.12909	0.00412	<0.001
Riots/protests	−0.10311	0.00467	<0.001
Battle: no-change of territory	−0.07233	0.00387	<0.001
Non-violent transfer of territory	−0.02687	0.00735	<0.001

The model trend component (defined by the conflicts variables and autoregressive term) explains 45.5 % of the overall variability, while 18.8 % is explained by the seasonality component and 23.9 % from the autocorrelation. Overall, the model explains 88.2 % of the variability, suggesting that other factors are influencing the present results. This can also be inferred by the strong relationship between *Pf*PR_*b*_ and Δ*P* (Table 2), the significant seasonality and autocorrelation, and the amount of model noise. The strong correlation between *Pf*PR_*b*_ and Δ*P* meant that part of the Δ*P* was explained by an intrinsic variability in *Pf*PR, which may be due to other factors, such as health systems, malaria interventions, climate variations, human displacement, etc., as found elsewhere [24, 29]. These factors can also affect the spatially (hidden trends) and especially non-spatially correlated variance (known as the nugget variance in geostatistics, or measurement error), which account for 11 % of the sill (the total amount of variance given by the sum of non-spatially correlated variance and spatially correlated variance in the residuals) [54]. On the other hand, the large amount of spatially correlated variance (the remaining 89 %) confirms the importance of spatiotemporal autocorrelation in malaria surveys [55] and conflict events [56] and in general for malaria transmission statistical analyses [11, 31, 42, 45, 57]. In this study, the optimization of the variogram based on the distances between conflicts and variance in the malaria surveys (Eq. 2), shows that conflicts were more likely to influence Δ*P* over short time periods (less than a year) and large spatial scales (up to 500 km). This indicates that malaria surveys closer to the conflicts are likely to have similar values, which are influenced by the type, duration and number of conflicts (the variables of the trend model component) and unknown factors. The differences between *Pf*PRs increase with the spatiotemporal distance from the conflicts, and the relative contribute of the conflict type and intensity to their variation is relatively lower. In this context, interventions rapidly deployed over short time periods especially in the less violent conflicts, can result in no-changes in *ΔP* or even its improving.

## Discussion

The period between 1997 and 2010 saw substantial numbers of armed conflicts across Africa, of varying length, intensity and type (Fig. 1a, b). Widespread evidence of the disruptive impacts of conflicts on malaria control efforts and transmission exist, but the impacts across large areas and over time have never been quantitatively explored. This research aimed to quantify the link between violence and increase in malaria.

With many African countries harbouring elimination ambitions and global eradication on the international agenda, understanding how much of a barrier conflicts place in terms of transmission changes becomes important. Analyses here show that, in general, locations affected by larger number of longer and closer conflicts with significant amounts of violence and deaths, are more likely to see an increase in *P. falciparum* prevalence. An example is Sierra Leone (increase in malaria transmission of 60 % compared to the level pre-conflict) for which the war from 1991 to 2001 was not only characterized by a large number of conflicts, but also by an unprecedented trail of atrocities [58]. However, the majority of the overall variability in *Pf*PR is not explained by the armed conflicts, but other factors such as seasonality, autocorrelation and the level of *Pf*PR before conflict events (which are probably a proxy for other variables not considered in this analysis) contribute to explain the changes in malaria transmission. This is the reason why for some of the most conflict-affected countries, such as Zimbabwe, Democratic Republic of Congo, and Somalia significantly lower *Pf*PR values were evident in the months after conflict events (or in Liberia, Mozambique, and Sudan no significant changes), compared to before [22, 59, 60]. In Sudan, for example, the impact of conflicts on malaria were balanced by disease surveillance, early warning and response systems implemented during the battles [17, 61].

Despite many of the most conflict-affected countries showing a decrease in *Pf*PR, others showed a strong increase. Guinea-Bissau, Cote d’Ivoire, Senegal, Sierra Leone, Guinea, Malawi, and Madagascar showed a large number of conflicts and increases in *Pf*PR due, in part, to the intervention coverage interruptions that followed [62]. Specifically, in Cote d’Ivoire (48 % increase in *Pf*PR), the 2002/2003 civil war [11] resulted in serious health system failures in the northern, western and central regions of the country, with more than 60 % of trained health personnel fleeing [63]. Moreover, lower levels of conflict were associated with increases in *P. falciparum* transmission. For example, in Burkina Faso (17 % increase in *Pf*PR), where most of the conflicts were associated with violence.

What lies behind the unexplained variation in *Pf*PR or hidden in other model components (*Pf*PR_b_, seasonality and autocorrelation)? Each region and conflict, of course, has its own unique conditions and drivers, but just as the relative effect size of the negative impacts of climate change on malaria can be dwarfed by those of control efforts [55], so it seems can post-conflict impacts. The burden of malaria in many African countries has declined substantially in the past decade [29, 64], coinciding with (1) the scaling up of malaria interventions supported by increased international funding for malaria control [29, 65, 66, 67], and (2) increasing urbanization and development [68]. In 2000, only 1.8 % African children slept under insecticide-treated nets (ITNs) in stable endemic areas, and this rose to 18.5 % by 2007 [69], and continues to rise today [29, 70]. Therefore, the changes in malaria transmission brought by expanding coverage of malaria intervention likely outweigh the negative impacts of armed conflicts over the timescales examined here. Moreover, while violent events undoubtedly result in misery and devastation for almost all involved, conflicts can often result in improved coordination and effort among key actors in the health field and bring more attention from humanitarian organizations [71, 72], producing a sustained impact that lasts beyond the ceasing of conflict. Finally, the longer term trends of rising urbanization and development all point towards sustained reductions in transmission [68], likely overriding any shorter term impacts of conflict. Resilience to the negative effects of conflict on malaria across much of Africa is evident, offering hope for the longer term prospects of control and elimination of the disease in the face of any future violence.

While clear patterns in the association between conflict and *P. falciparum* malaria exist, limitations in the analyses presented do remain. The short temporal range found after adjusting for seasonality may be due to the variability of intervention and control measures in place [46], which can introduce noise in the temporal dependence between conflict events and malaria surveys and, therefore, shorten the temporal influence of conflicts on *ΔP*. In addition, the seasonal model is global and hence local variations (country by country) may have been smoothed as well as not taking into consideration local climate factors. For example, climate anomalies can affect: (1) the timing and duration of the transmission season which influences both the malaria prevalence and planned interventions [48, 73], and (2) the level of conflict and risk of violence in an area [41]. If available, sample weights could help in improving the accuracy of the association between conflict variables and changes in malaria prevalence; however, at the inferential stage their effects are limited by the use of thousands of conflict-to-malaria point associations. Additionally, data quality represents a potential source of uncertainty because, in conflict situations, it can be difficult to collect reliable data on malaria prevalence and transmission [22, 74], though here the analysis is applied to pre- and post-conflict prevalence at same locations, so this issue is limited. In terms of conflict data, there are alternative databases, but each has their own limitations for a point-based spatiotemporal analysis. For example, the Uppsala database [75] contains less data on conflicts than ACLED; the HIIK conflict barometer [76] and the EM-DAT [77] do not provide conflict information with the same spatial resolution as ACLED [40]. Moreover, a more detailed local study could utilize other non-public sources of data, such as humanitarian funding appeals. Nevertheless, it has been demonstrated that ACLED likely represents the most reliable dataset for continental and national point data analyses [41, 78, 79]. However, while all types of conflict were significantly associated in space and time with *Pf*PR variations, their impact is certainly likely to be different [59], and also the database does not account for other politically complex emergencies [61] that are not recognized as conflicts. Finally, a key missing component in this analysis is the underlying population mobility, which if data were available, could improve the accuracy of this analysis [80], since it affects not only malaria prevalence, but also health infrastructure through movements of refugees [24, 81]. In fact, it is likely that temporary migratory movements of people escaping from conflicts have concentrated malaria-affected people in conflict-free areas and exposed refugees to malaria when they escape to rural, high-endemicity areas [81–84]. This may have contributed to the positive fixed effect coefficient for the *Pf*PR_b_ (the autoregressive term) in which high levels of prevalence before conflict are associated with high positive differences, and on the spatiotemporal autocorrelation noise (the nugget effect), but without reliable and consistent continent-wide data on refugee movements, it was not possible to account for them in this analysis.

## Conclusion

This analysis shows that the gains made against malaria over the past decade can be maintained in even the most difficult of political and health circumstances [57, 85, 86, 87]. For many parts of Africa, the concept that conflicts threaten malaria elimination aspirations in the long term may be another myth to add to those that block progress for the poor [88]. Conflicts are one important, but not the major component, in determining the *Pf*PR post-conflicts; therefore, in presence of conflicts, in most places malaria prevalence keep decreasing from pre-conflict level. However, the impact of the conflicts on malaria prevalence is stronger in the presence of violent events (e.g., violence against civilians and riots/protests). Although further analyses are needed to understand the mechanism by which violence influences malaria, this research showed the need for tackling the difficult task of maintaining intervention coverage in settings that are both under conflict and still suffering from high *P. falciparum* burden.

The world is becoming a more peaceful place [89], but further conflicts in endemic-malaria zones are inevitable. While such disruptive events may divert attention and resources away from malaria control and elimination efforts temporarily, they need not effect permanent or long-term damage to prospects for a malaria-free world.
